# The Aggravation of Neuropsychiatric Symptoms in the Offspring of a Korean Family with Intellectual Disability and Developmental Delay Caused by a Novel *ARX* p.Lys385Ter Variant

**DOI:** 10.3390/ijms251910327

**Published:** 2024-09-25

**Authors:** Ji Yoon Han, Tae Yun Kim, Jin Gwack, Joonhong Park

**Affiliations:** 1 Department of Pediatrics, College of Medicine, The Catholic University of Korea, Seoul 06591, Republic of Korea; han024@catholic.ac.kr; 2 Department of Thoracic and Cardiovascular Surgery, Jeonbuk National University Medical School and Hospital, Jeonju 54907, Republic of Korea; cseokim@jbnu.ac.kr; 3 Department of Preventive Medicine, Jeonbuk National University Medical School, Jeonju 54907, Republic of Korea; 4 Research Institute of Clinical Medicine of Jeonbuk National University-Biomedical Research Institute of Jeonbuk National University Hospital, Jeonju 54907, Republic of Korea; 5 Department of Laboratory Medicine, Jeonbuk National University Medical School and Hospital, Jeonju 54907, Republic of Korea

**Keywords:** Aristaless-related homeobox, X linked, *ARX*, intellectual developmental disorder, X linked 29, developmental epileptic encephalopathy, agenesis of the corpus callosum, clinical exome sequencing

## Abstract

The *ARX* mutations encompass a nearly continuous spectrum of neurodevelopmental disorders (NDDs), ranging from lissencephaly to Proud syndrome, as well as infantile spasms without brain malformations, and including both syndromic and non-syndromic intellectual disabilities (IDs). We describe worsening neuropsychiatric symptoms in the offspring of a Korean family with ID/developmental delay (DD) caused by a novel *ARX* p.Lys385Ter variant. Sequential genetic testing was performed to investigate the ID, DD, agenesis of the corpus callosum (ACC), and developmental epileptic encephalopathy (DEE) observed in the proband. A comprehensive trio clinical exome sequencing approach using a Celemics G-Mendeliome Clinical Exome Sequencing Panel was employed. Given the clinical manifestations observed in the proband, gene panel sequencing identified a heterozygous *ARX* variant, c.1153A>T/p.Lys385Ter (Reference transcript ID: NM_139058.3), as the most likely cause of ID, DD, ACC, and DEE in the proband. Sanger sequencing confirmed the segregation of the *ARX* variant, c.1153A>T/p.Lys385Ter, with the phenotype and established the maternally inherited dominant status of the heterozygous variant in the patient, as well as in her grandmother, mother, and aunt. Our case report adds to the understanding of the female phenotype in *ARX*-related disorders caused by loss-of-function variants in the *ARX* gene. Genetic counseling for *ARX* families should proceed with caution, as female carriers can exhibit a wide range of phenotypes, from normal cognitive development to ID/DD, ACC, and DEE.

## 1. Introduction

The *ARX* gene, located at Xp.21.3, encodes for the Aristaless-related homeobox protein, a member of the Aristaless-related group within the paired class of homeodomain (HD) proteins. The ARX protein is crucial for brain development, playing a role in the differentiation and tangential migration of GABAergic interneurons, as well as the development of excitatory cholinergic neurons [1,2]. The *ARX* mutations encompass a nearly continuous spectrum of neurodevelopmental disorders (NDDs), including developmental and epileptic encephalopathy (DEE) 1 (MIM #308350), hydranencephaly with abnormal genitalia (MIM #300215), intellectual developmental disorder, X-linked 29 (MIM #300419), lissencephaly, X-linked 2 (MIM #300215), Partington syndrome (MIM #309510), and Proud syndrome (MIM #300004). A single recessive gene mutation on the X chromosome can cause X-linked recessive diseases. Because X-linked recessive conditions most frequently affect males, those carrying pathogenic *ARX* variants typically exhibit the disease symptoms. To date, more than 100 male patients with pathologic loss-of-function (LoF) variants in the *ARX* gene have been reported, and the associated phenotype is characterized by constant intellectual disability (ID) that can be associated with neuronal migration defects and severe epilepsy. Although *ARX* mutations tend to have a more severe impact on males, female carriers of the mutations can also be mildly affected or usually unaffected [3,4]. Several cases of truncate and missense mutations in *ARX* have shown severe phenotypes similar to those observed in males [5,6]. In contrast, females carrying *ARX* pathogenic variants have usually been described as asymptomatic or with a mild phenotype [7]. An Australian family with multiple affected individuals, including males with lissencephaly, X-linked with abnormal genitalia (XLAG) syndrome and two females with ID and epilepsy, has been documented [8]. Female carriers reported in familial studies are typically asymptomatic or present with mild phenotypes. However, sporadic cases of severe phenotypes due to de novo *ARX* variants have been documented in a few studies. [9]. Recently, a review of the phenotypes of females with *ARX* variants from the literature has provided new insights into the clinical spectrum and variability of these conditions [10]. The clinical spectrum in females carrying *ARX* variants is broad, ranging from asymptomatic forms to severe NDDs. Approximately one-fifth of affected females present with isolated agenesis of the corpus callosum (ACC) or mild symptoms such as learning disabilities, autism spectrum disorder (ASD) without ID, or drug-responsive epilepsy. Notably, the ID/DEE phenotype is significantly more prevalent in women with de novo *ARX* variants compared to those with inherited variants. This higher prevalence of severe phenotypes among individuals with de novo variants can be attributed to the impact on reproductive fitness; individuals with severe phenotypes are less likely to reproduce, leading to these severe cases occurring more frequently due to new mutations.

In this study, we describe the aggravation of neuropsychiatric symptoms in the offspring of a Korean family with ID and DD caused by a novel *ARX* p.Lys385Ter Variant.

## 2. Case Presentation

### 2.1. The Proband

**Figure 1:**
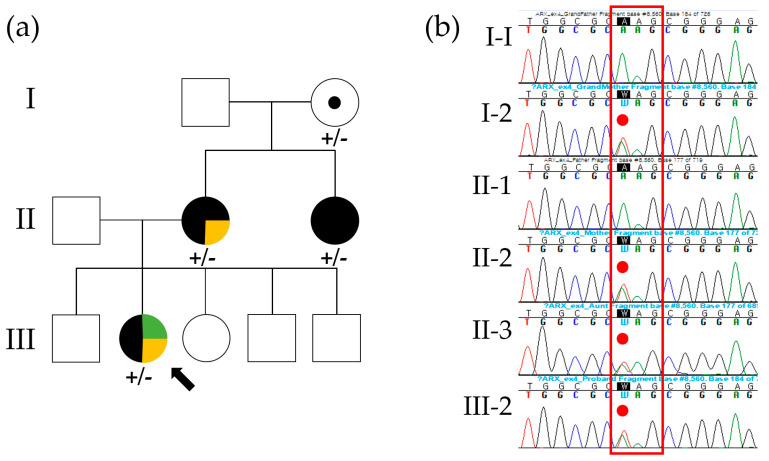
Pedigree and segregation analysis of the *ARX* c.1153A>T/p.Lys385Ter variant in a Korean family. (**a**) In the provided pedigree, the black-filled symbol represents individuals with developmental delay and/or intellectual disability. The yellow-filled symbol indicates individuals with dysgenesis of the corpus callosum. The green-filled symbol denotes individuals with epilepsy. The black arrow indicates the proband. +, *ARX* c.1153A>T/p.Lys385Ter variant; -, wild-type. (**b**) The *ARX* c.1153A>T/p.Lys385Ter variant, indicated by a red dot, was identified as heterozygous in the proband’s grandmother, mother, aunt, and the proband through segregation analysis. The three bases constituting the 385th codon are marked with red box.

**Figure 2:**
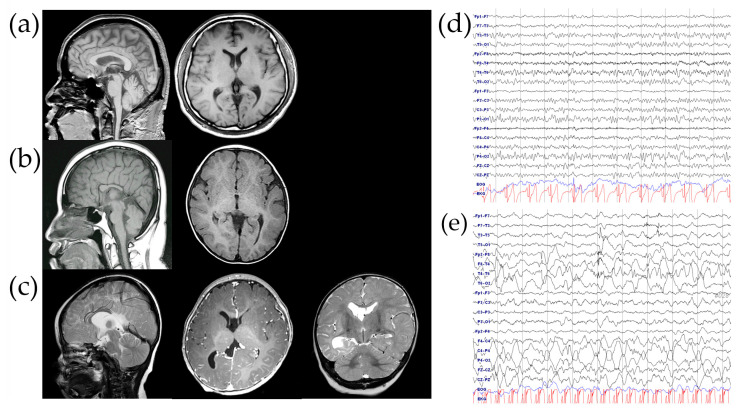
The results of brain magnetic resonance imaging (MRI) and an electroencephalogram (EEG). (**a**–**c**) A brain MRI exhibited normal findings in the proband’s grandmother (**a**); a hypoplastic corpus callosum with absent rostrum, splenium, and posterior body in the proband’s mother (**b**); and dysgenesis of the corpus callosum with migration abnormality in the proband. (**d**,**e**) The EEG showed normal findings in the proband’s mother (**d**) but abnormal EEG findings, with sharp and slow waves in the right temporo-occipital areas in the proband (**e**).

The proband (III-2 in Figure 1a) is a female born to non-consanguineous parents. She was delivered vaginally at 36 weeks of gestation with normal growth parameters: a birth weight of 3 kg (30th percentile), a height of 51 cm (84th percentile), and a head circumference of 35 cm (83rd percentile). There were no complications noted during the newborn period. Seizures began at 5 months of age, characterized by sudden blank staring and circumoral cyanosis lasting a few seconds. Her seizures included generalized tonic–clonic, absence, and focal types. She is currently being treated with valproic acid and levetiracetam and has been seizure-free for 2 years. Although her somatic growth was normal, she exhibited DD. She achieved head control by 3 months, was able to sit unsupported by 9 months, and walked independently by 18 months. At 2.5 years, her language development was delayed, as she spoke only a few words. An assessment using the Bayley Scales of Infant and Toddler Development, Third Edition (Bayley-III), at age 3 revealed global DD, with cognitive, motor, and language developmental ages of 18 months, 25 months, and 13 months, respectively. Her four siblings (three males and one female) are healthy, with no history of ID, DD, or epilepsy. Physical examination of the proband revealed no abnormalities, including no facial dysmorphism or congenital malformations. Cardiac, pulmonary, gastrointestinal, and genitourinary examinations were unremarkable. Visual and auditory evoked potential tests were normal. Routine laboratory tests and metabolic assessments, including plasma lactate, pyruvate, ammonia, urine organic acids, plasma acylcarnitine, and plasma amino acids, were all within normal limits. Brain magnetic resonance imaging (MRI) demonstrated ACC, abnormal migration, and enlargement of the posterior right ventricle (Figure 2c). Electroencephalography (EEG) showed focal sharp and slow waves in the right temporo-occipital areas (Figure 2e). Over the course of a year, she experienced recurrent seizures and was resistant to multiple anti-seizure medications. However, with ongoing treatment with valproic acid and levetiracetam, she has been seizure-free for the past 2 years.

### 2.2. The Proband’s Mother

The proband’s mother (II-2 in Figure 1a) was born to non-consanguineous parents at 38 weeks of gestation following an uncomplicated pregnancy, with a birth weight of 3.5 kg (70th percentile). She completed general middle school with special education support but did not pursue employment afterward, becoming a full-time housewife after marriage. An intelligence quotient (IQ) test administered at age 7 showed a score of 65, indicating mild intellectual impairment and a need for assistance with complex tasks. She has no history of seizures or other physical or medical problems. A brain MRI conducted at age 25 revealed dysgenesis of the corpus callosum (Figure 2b), while EEG showed a normal background pattern without epileptiform discharges (Figure 2d). She became obese during middle school, with a body mass index of 34 kg/m^2^ at age 25. Metabolic tests, including fasting blood glucose, thyroid function, and lipid profiles, were all within normal ranges. She has remained in good health to date and has shown no medical issues.

### 2.3. The Proband’s Grandmother

The proband’s grandmother (I-2 in Figure 1a) was born at term via normal vaginal delivery following an uneventful pregnancy. During her pediatric years, she was in good health with no history of seizures or neurodevelopmental disorders. Her childhood IQ score was 75 (4.7th percentile), placing her in the borderline normal range. After graduating from elementary school, she worked as a shop clerk and later became a full-time housewife after marriage. A brain MRI conducted at age 30 showed a normal gyral pattern with no evidence of corpus callosum dysgenesis (Figure 2a).

## 3. Genetic Diagnosis

**Figure 3:**
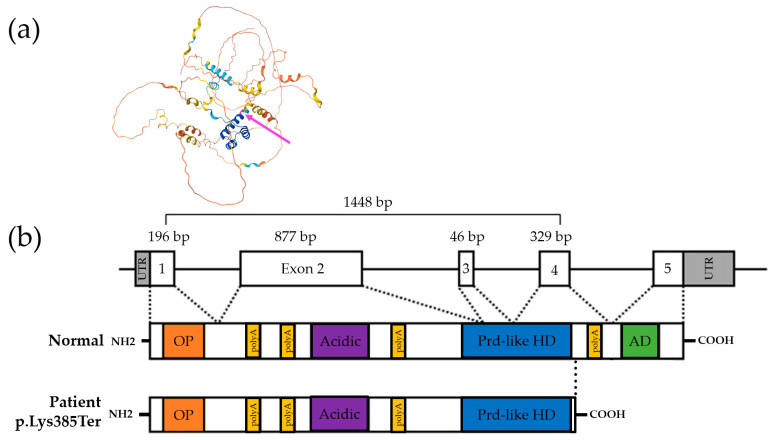
A schematic representation of the *ARX* gene and the predicted ARX protein. (**a**) Protein structure analysis using AlphaFold showed a very high per-residue confidence score (pLDDT) of 90.68 for the *ARX* p.Lys385 residue, highlighted in pink and indicated by a pink arrow. (**b**) The genomic structure of the *ARX* gene is shown, along with the approximate protein domains and key functional regions, including the octapeptide domain (OP), polyalanine tract (polyA), acidic domain (Acidic), prd-like homeodomain (prd-like HD), and Aristaless domain (AD). The numbers above the exons indicate their sizes in base pairs (bp). In the patient, the predicted ARX protein is truncated and lacks the 4th polyA and the AD.

Sequential genetic testing was performed to investigate the ID, DD, ACC, and DEE observed in the proband. Initially, conventional karyotyping and chromosomal microarray analysis were performed, but no pathogenic structural or numerical chromosome changes or copy number variations were identified. To further explore potential genetic causes, we utilized a comprehensive trio clinical exome sequencing (CES) approach, employing the Celemics G-Mendeliome Clinical Exome Sequencing Panel (Celemics, Inc., Seoul, Republic of Korea) on the proband and her parents. This panel includes a broad spectrum of 7000 genes linked to clinically significant Mendelian genetic diseases. Massively parallel sequencing was performed using a DNBSEQ-G400RS High-throughput Sequencing Set and DNBSEQ-G400 sequencer (MGI Tech Co. Ltd., Shenzhen, China). Pathogenic variants were interpreted according to the standards and guidelines of the American College of Medical Genetics and Genomics (ACMG) and the Association for Molecular Pathology (AMP). The filtering criteria for identifying potential harmful variants were as follows: (1) variants located near or within the exons of protein-coding genes associated with Mendelian diseases; (2) variants with allele frequencies below 0.01; (3) variants causing nonsynonymous or nonsense changes in exons, altering highly conserved splice sites, or inducing frameshift mutations; (4) de novo or rare heterozygous, compound heterozygous, or homozygous variants of the same gene identified in the proband; (5) genes associated with the clinical significance of the epilepsy panel (listed in Supplementary Table S1); (6) the specific phenotypes (ID, DD, ACC, and DEE) are considered either sporadic or inherited in a dominant manner, given that the proband’s mother and aunt were affected. The allele frequencies of filtered variants were estimated using the Genome Aggregation Database (gnomAD, https://gnomad.broadinstitute.org; accessed on 1 March 2024). In addition, in silico analysis was conducted to predict the pathogenicity of nonsynonymous and insertion/deletion variants using BayesDel addAF (https://varsome.com; accessed on 1 March 2024), MutationTaster (https://www.mutationtaster.org; accessed on 1 March 2024), and VEST-4 (https://www.cravat.us/CRAVAT/; accessed on 1 March 2024). As a result, gene panel sequencing identified a heterozygous *ARX* variant, c.1153A>T/p.Lys385Ter, as the best candidate for causing the ID, DD, ACC, and DEE in the proband (Reference transcript ID: NM_139058.3). This nonsense variant was absent from the Genome Aggregation Database (gnomAD) and was computationally predicted to be “disease-causing” by MutationTaster, “deleterious” by VEST-4 with a VEST score of 0.961 (*p* value of 0.01062), and “pathogenic very strong” by BayesDel addAF with a score of 0.7364. VEST-4 assigns variants a score between 0 and 1, with 1 indicating a confident prediction of a functional mutation. BayesDel addAF is a deleteriousness prediction meta-score that integrates various predictive factors, ranging from −1.11707 to 0.750927, with higher scores indicating a greater likelihood that the variant is pathogenic. No potential splice effect was detected by SpliceAI (https://spliceailookup.broadinstitute.org; accessed on 1 March 2024). The clinical presentation of the patient was consistent with ID, DD, ACC, and DEE caused by the *ARX* variant. Sanger sequencing confirmed the segregation of the *ARX* variant, c.1153A>T/p.Lys385Ter, with the phenotype and established the maternally inherited dominant status of the heterozygous variant in the patient, as well as in her grandmother, mother, and aunt (Figure 1b). Protein structure analysis using AlphaFold (https://alphafold.ebi.ac.uk/, accessed on 20 September 2024) showed high per-residue confidence scores (90 > pLDDT > 70) of 90.68 for the *ARX* p.Lys385 residue in the dimerization domain (Figure 3a). This variant was classified as likely pathogenic according to ACMG guidelines, based on the following criteria: PVS1, Null variant (nonsense, frameshift, canonical +/−1 or 2 splice sites, initiation codon, single- or multi-exon deletion) in a gene in which LoF is a known mechanism of disease; and PM2, Absent from controls (or present at an extremely low frequency if recessive) in Exome Sequencing Project, 1000 Genomes, or ExAC (Figure 3b).

## 4. Discussion

The *ARX* gene encodes a transcription factor that binds to the specific sequence motif 5′-TAATTA-3′ within the regulatory elements of target genes, including histone demethylase lysine-specific demethylase 5C (*KDM5C*) [11]. It positively regulates the transcription of *KDM5C* and synergistically activates its expression alongside the histone lysine demethylase *PHF8*, potentially competing with the transcription regulator *ZNF711* [12]. This synergy may be linked to the enrichment of histone H3K4me3 in regulatory elements. The *ARX* gene is essential for normal brain development, playing a crucial role in neuronal proliferation, interneuronal migration, and differentiation in the embryonic forebrain [3,13]. The *ARX* gene belongs to the group-II Aristaless-related protein family, which is expressed primarily in the central and/or peripheral nervous systems. Mutations, including polyalanine tract expansions, are associated with X-linked cognitive impairment and epilepsy [14]. ARX expression is prominent in neuronal precursors within the germinal matrix and ventricular zones throughout all stages of development [15]. High expression levels are also noted in the subventricular zone, caudate nucleus, putamen, substantia nigra, corpus callosum, amygdala, and hippocampus [16,17]. The *ARX* gene plays a crucial role in early embryonic development, regulating brain structure formation and being involved in neuronal proliferation, interneuronal migration, and differentiation [18,19]. The ARX protein includes several functional domains: an octapeptide domain, a homeodomain, four polyalanine tracts, three nuclear localization sequence motifs, and a C-terminal Aristaless domain [20]. Pathogenic variants in *ARX* have pleiotropic effects, with clinical presentations ranging from mild ID to DEE with brain anomalies [21]. Clinical manifestations vary widely and can include DD/ID, learning disabilities, behavioral disorders, autism spectrum disorders, dystonia, and various types of seizures, such as infantile spasms, myoclonic seizures, and tonic–clonic seizures. Expansions and duplications of the first or second polyalanine tracts can result in non-malformation phenotypes, including epilepsy and dystonic movement disorders [3,22,23,24]. Truncating mutations and certain missense mutations lead to syndromic forms with brain malformations, including lissencephaly, midbrain malformations, and ACC [4,5,22,25,26,27]. XLAG is associated with mutations that cause either premature protein truncation or missense mutations in highly conserved residues within the homeobox domain [28].

Females carrying *ARX* pathogenic variants are often described as asymptomatic or exhibiting only mild phenotypes [29]. While some LoF variants in males can lead to severe malformation phenotypes, which are often lethal within the first months of life, female relatives may display a milder phenotype [8,26]. The affected individuals are predominantly male, but female phenotypes, typically identified through the study of mothers or other female relatives of male probands, include abnormalities in the corpus callosum and sometimes mild ID, potentially associated with epilepsy and/or psychiatric conditions such as anxiety, depression, and schizophrenia [5,26]. Recent research by Gras et al. provides a comprehensive overview of the clinical features in female carriers. Their study revealed that 42.5% of female carriers are asymptomatic and 16.4% exhibit isolated ACC or mild symptoms such as learning difficulties, ASD, and epilepsy without ID, while 41% present with severe phenotypes [10]. Among the patients, 42 (58%) carried truncating variants, 31 (41%) had missense variants, and 1 (1%) had an in-frame deletion. Severe phenotypes were more common in carriers with truncating mutations (52%) compared to those with missense variants (26%) and were also more frequent in de novo cases (75%) than in inherited cases (28%). Abnormalities of the corpus callosum are highly penetrant in female carriers of *ARX* variants, reported in 67% of patients. NDDs are observed in approximately half of the female carriers, and epilepsy occurs in about one-third. A Chinese study found that female carriers of *ARX* mutations have more than a fourfold increased risk of non-syndromic ID [30]. ACC, a malformation commonly reported in affected males, is also observed in asymptomatic females. ACC is a highly penetrant trait among female carriers of *ARX* variants, being present in two-thirds of the patients studied [10]. While ACC is more frequent in those with ID/DEE, it is also found in individuals without any NDDs. Therefore, ACC should not be considered a definitive predictor of the neurodevelopmental phenotype due to potential bias. Asymptomatic carriers often do not undergo routine brain MRI, which can prevent the identification of ACC in this group. Previous reports have documented similar intra-familial variability, ranging from normal to severe phenotypes [3,26,31]. Due to its X-linked inheritance pattern, the expression of the disease can vary significantly among females. In this family, the female subjects exhibit truncating mutations, which may lead to more severe symptoms. Clinical manifestations range from normal phenotypes to DEE, with brain MRI scans revealing a spectrum of abnormalities, including variations in the corpus callosum and myelination. While this may be coincidental, there appears to be a worsening of symptoms across generations, suggesting that this observation warrants reporting. In this study, intra-familial variability was observed, ranging from a normal phenotype in the proband’s grandmother and mild ID with partial dysgenesis of the corpus callosum in the proband’s mother to DEE with dysgenesis of the corpus callosum and migration abnormalities in the proband. The proband exhibits a phenotype consistent with DEE 1 (MIM #308350), while the proband’s mother is considered to have intellectual developmental disorder, X-linked 29 (MIM #300419). However, the proband’s grandmother presents as normal and is classified as an asymptomatic female carrier. Given the nature of X-linked diseases in females, where a single mutation can lead to a wide range of clinical manifestations, it is challenging to classify these cases into a single category. Clinical characteristics in a Korean family with ID/DD caused by a novel *ARX* p.lys385Ter variant are summarized in Table 1.

The differences in the effects of X-linked pathogenic variants between sexes are attributed to X-inactivation, the mechanism of X-chromosome dosage compensation [32]. One hypothesis for the variability in the clinical expression of *ARX*-related disorders in women is that skewed X-chromosome inactivation (XCI) may contribute to phenotypic differences, as the *ARX* gene undergoes XCI. Studies of XCI have shown no significant bias in blood cells for some patients [6,8,10,33]. However, the published research indicates that XCI patterns can vary both between individuals and within individuals across different cell types [7,29,34]. Since *ARX*-related symptoms are caused primarily by LoF variants affecting the brain, it is anticipated that the severity of the phenotype may be more closely related to the XCI pattern in brain tissue rather than in the blood. Therefore, to better understand the susceptibility of *ARX*-related disorders in females, it is essential to conduct extensive XCI testing in female carriers across various tissues. Genetic variants in other genes can influence *ARX*-related phenotypes. For instance, a regulatory pathway associated with X-linked ID and epilepsy links *KDM5C* to polyalanine expansions in *ARX*, suggesting that the length of polyalanine tracts may correlate with the severity of X-linked ID and/or epilepsy [35]. Additionally, other factors, such as epigenetic modifications and environmental influences, also contribute to phenotypic variability. The interaction between these factors could potentially have a more significant impact on the phenotype than X-inactivation patterns in the brain alone. Despite the existing information on genotype–phenotype correlations, the extent of intra- and inter-familial variability in the expression of *ARX* mutations remains uncertain. Accurate phenotypic information is crucial for genetic counseling in *ARX* families and highlights the importance of considering prenatal diagnosis for at-risk female relatives. This report enhances the understanding of the pathogenic role of the *ARX* gene in female carriers and advocates for the molecular analysis of *ARX* in female patients to better establish genotype–phenotype correlations.

## 5. Conclusions

In conclusion, our case report adds to the understanding of the female phenotype in *ARX*-related disorders caused by loss-of-function variants in the *ARX* gene. Genetic counseling for *ARX* families should proceed with caution, as female carriers can exhibit a wide range of phenotypes, from normal cognitive development to ID/DD and DEE. Additionally, this study confirms the association between ACC and *ARX* variants in women, although ACC alone may not accurately reflect the underlying cognitive function. This information is crucial for genetic counseling in *ARX* families and suggests considering prenatal diagnosis for at-risk female fetuses.

## Figures and Tables

**Table 1:** Summary of Clinical Characteristics in a Korean Family with Intellectual Disability/Developmental Delay Caused by a Novel *ARX* p.Lys385Ter Variant.

	Grandmother (I-2)	Mother (II-2)	Aunt (II-3)	The Proband (III-2)
**Cognitive ability**				
DD/ID	Absent	Mild ID	Mild ID	Severe DD
ASD	Absent	Absent	Absent	Absent
Learning disability	Present	Present	Present	Present
**Brain MRI**				
ACC	Absent	Present	Not assessed	Present
Gyration abnormalities	Absent	Absent	Not assessed	Present
**Epilepsy**				
Age at first seizures	Absent	Absent	Absent	5 months
Initial epileptic features	Absent	Absent	Absent	Generalized seizures
Epilepsy	Absent	Absent	Absent	Present
DEE	Absent	Absent	Absent	Present
Pharmacoresistance	Absent	Absent	Absent	Present *
Epileptic manifestation at last examination	Absent	Absent	Absent	Controlled with polytherapy

ID, intellectual disability; DD, developmental delay; ASD, autism spectrum disorder; MRI, magnetic resonance imaging; ACC, agenesis of the corpus callosum; DEE, developmental and epileptic encephalopathy. * Initially, controlling the seizures was difficult; however, two years later, the seizures were well managed with polytherapy.

## Data Availability

The data are contained within the article.

## Supplementary Materials

The following supporting information can be downloaded at: https://www.mdpi.com/article/10.3390/ijms251910327/s1.
