# Supplementary material for: The Aggravation of Neuropsychiatric Symptoms in the Offspring of a Korean Family with Intellectual Disability and Developmental Delay Caused by a Novel ARX p.Lys385Ter Variant
Source: Int J Mol Sci. 2024 Sep 25;25(19):10327. doi: 10.3390/ijms251910327 (PMC11476583; doi:10.3390/ijms251910327)
Supplement: Supplementary file 1 [file ijms-25-10327-s001.zip › ijms-3189333-supplementary.pdf]

Supplementary Table S1. 652 genes included in the hereditary epilepsy panel

AARS1, ABAT, ACOX1, ACTL6B, ADAR, ADGRG1, ADPRS, ADSL, AFF3, AIMP1, AKT3, ALDH5A1, ALDH7A1, ALG1, ALG11, ALG13, ALG3, ALG6, ALG8, ALG9, ALPL, AMPD2, AMT, AP2M1, AP3B2, ARFGEF2, ARG1, ARHGEF9, ARID1B, ARV1, ARX, ASAH1, ASPA, ATN1, ATP1A1, ATP1A2, ATP1A3, ATP6V0A2, ATP6V1A, ATP7A, ATRX, BCKDHA, BCKDHB, BCS1L, BOLA3, BRAF, BRAT1, BSCL2, BTD, C12orf57, CACNA1A, CACNA1B, CACNA1D, CACNA1E, CACNA1G, CACNA2D2, CAD, CASK, CC2D2A, CDKL5, CHD2, CHRNA2, CHRNA4, CHRNB2, CIC, CLCN4, CLN3, CLN8, CLTC, CNKSR2, CNNM2, CNPY3, CNTNAP2, COG7, COL18A1, COL4A1, COL4A2, COQ2, COQ4, COQ9, CPA6, CREBBP, CSNK2B, CSTB, CTNNA2, CTSD, CUL4B, CUX2, CYFIP2, D2HGDH, DBT, DCX, DDX3X, DEAF1, DEGS1, DENND5A, DEPDC5, DHDDS, DHPS, DHX30, DIAPH1, DNM1, DNM1L, DOCK7, DPAGT1, DPM1, DPYD, DYNC1H1, DYRK1A, EARS2, EEF1A2, EFTUD2, EHMT1, EIF2B1, EIF2B2, EIF2B3, EIF2B4, EIF2B5, EIF2S3, EIF3F, EML1, EPG5, EPM2A, ETHE1, EXOSC3, FARS2, FBXL4, FBXO11, FGF12, FKTN, FLNA, FOLR1, FOXG1, FRRS1L, FUCA1, FUT8, GABBR2, GABRA1, GABRA2, GABRA5, GABRB2, GABRB3, GABRG2, GALC, GAMT, GBA, GFAP, GLB1, GLDC, GLUD1, GLUL, GM2A, GNAO1, GNAQ, GNB1, GNB5, GOSR2, GOT2, GPAA1, GPHN, GRIA4, GRIN1, GRIN2A, GRIN2B, GRIN2D, GTPBP2, HACE1, HAX1, HCFC1, HCN1, HCN2, HECW2, HEPACAM, HEXA, HEXB, HMGCL, HNRNPH2, HNRNPR, HNRNPU, HRAS, HSD17B4, HTRA2, IER3IP1, IFIH1, IKBKG, IQSEC2, IRF2BPL, ITPA, KARS1, KCNA2, KCNB1, KCNC1, KCNJ10, KCNJ11, KCNK4, KCNMA1, KCNQ2, KCNQ3, KCNQ5, KCNT1, KCNT2, KCTD3, KCTD7, KIAA1109, KIF1A, KIF2A, KIF5C, KMT2E, KRAS, LGI1, LIAS, MACF1, MAF, MAGI2, MAP2K1, MAP2K2, MBD5, MBOAT7, MDH2, MECP2, MED12, MEF2C, MFF, MFSD8, MLC1, MMACHC, MMADHC, MOCS1, MOCS2, MOGS, MPDU1, MTHFR, MTOR, NACC1, NAGA, NARS2, NBEA, NDE1, NDUFA1, NDUFA10, NDUFAF2, NDUFAF5, NDUFS4, NDUFS8, NDUFV1, NEXMIF, NGLY1, NHLRC1, NPRL2, NPRL3, NRXN1, NSD1, NSDHL, NTRK2, NUS1, OCLN, OPHN1, OTUD6B, P4HTM, PACS1, PACS2, PAFAH1B1, PAH, PAK1, PARS2, PCCA, PCCB, PCDH12, PCDH19, PDHA1, PDHX, PET100, PHACTR1, PHGDH, PIGA, PIGB, PIGC, PIGG, PIGH, PIGN, PIGO, PIGQ, PIGT, PIGU, PIGW, PIK3R2, PLAA, PLCB1, PLPBP, PMM2, PNKP, PNPO, POLG, POMGNT1, POMT1, PPP2CA, PPP3CA, PPT1, PRICKLE1, PRMT7, PRRT2, PSAP, PTPN23, PTS, PURA, QARS1, RAB11B, RAB18, RALA, RARS2, RELN, RFT1, RHOBTB2, RMND1, RNASEH2A, RNASEH2B, RNASEH2C, RNASET2, ROGDI, RORA, RORB, RTN4IP1, RTTN, SAMHD1, SCARB2, SCN1A, SCN1B, SCN2A, SCN3A, SCN8A, SEPSECS, SETBP1, SGSH, SIK1, SLC12A5, SLC13A5, SLC16A2, SLC1A2, SLC1A4, SLC25A1, SLC25A12, SLC25A22, SLC2A1, SLC35A2, SLC6A1, SLC6A8, SLC9A6, SMARCA2, SMARCC2, SMC1A, SMS, SNAP25, SNORD118, SPATA5, SPTAN1, ST3GAL5, STAG1, STAMBP, STRADA, STX1B, STXBP1, SUCLA2, SUOX, SURF1, SYN1, SYNGAP1, SYNJ1, SZT2, TANGO2, TBC1D24, TBCD, TBCK, TBL1XR1, TCF4, TDP2, TPP1, TRAK1, TREX1, TRIM8, TRPM6, TSC1, TSC2, TSEN54, TUBA1A, TUBB2A, TUBB2B, TUBB3, TUBB4A, TUBG1, UBA5, UBE2A, UBE3A, UFM1, UNC80, VAMP2, VARS1, VPS11, WARS2, WASF1, WDR37, WDR45, WDR73, WWOX, YWHAG, ZBTB18, ZDHHC9, ZEB2, ZNF142, ABCA2, ADAM22, ADARB1, ADAT3, AIMP2, ALG14, ALKBH8, ANKRD11, APC2, ASNS, ATP5F1A, ATP6AP2, CACNA1H, CACNB4, CARS2, CCDC88A, CCDC88C, CDC42BPB, CDK19, CELF2, CEP85L, CERS1, CLN6, CLPB, COG4, COG6, COG8, COQ6, COX10, COX15, CPLX1, CRPPA, CTSF, CTU2, CUL3, CYP27A1, DDC, DHCR24, DHCR7, DHX16, DLL1, DMXL2, DNAJC5, DNAJC6, DOLK, DPM2, EFHC1, EIF2AK2, EMC10, EMX2, EXOC7, EXT2, FAM50A, FAR1, FASTKD2, FBXO28, FCSK, FDFT1, FGF13, FGFR3, FH, FKRP, FOXRED1, GABRB1, GAD1, GALNT2, GCH1, GFM1, GLI3, GLRA1, GLS, GLYCTK, GRIA2, GRM7, GRN, GSS, GTPBP3, GUF1, H3-3A, H3-3B, HCCS, HEATR5B, HERC2, HLCS, HOXA1, HPDL, HPRT1, HSPD1, KAT5, KAT8, KATNB1, KCNA1, KCNH1, KIFBP, KPTN, LARGE1, LARS1, LIPT1, LIPT2, LMAN2L, LMBRD2, LMNB1, LNPK, LSS, LYST, MADD, MANBA, MAST1, MED17, MED27, MINPP1, MTHFS, MTR, NARS1, NCDN, NDP, NDUFA2, NDUFAF3, NDUFAF4, NDUFS1, NDUFS2, NDUFS6, NDUFS7, NECAP1, NEDD4L, NEUROD2, NR4A2, NRROS, NSF, NUBPL, NUP214, OTX2, OXR1, PCDHGC4, PCLO, PCYT2, PDSS2, PEX1, PEX10, PEX12, PEX13, PEX19, PEX2, PEX3, PEX5, PEX6, PEX7, PIGK, PIGP, PIGS, PLA2G6, PMPCB, PNPT1, POLG2, POMT2, PPIL1, PRODH, PSAT1, PSPH, PTF1A, PUM1, QDPR, RAB3GAP1, RAB3GAP2, RALGAPA1, RALGAPB, RANBP2, RARS1, RNF113A, RNF13, RNF2, RNU4ATAC, RPIA, RRM2B, RUSC2, RYR2, SAMD12, SATB1, SATB2, SCAF4, SCAMP5, SCO1, SCO2, SDHA, SEMA6B, SERPINI1, SETD1A, SETD1B, SETD5, SIX3, SLC35A1, SLC35A3, SLC39A8, SLC45A1, SLC5A6, SNIP1, SNX27, SPR, SPTBN1, SPTBN4, ST3GAL3, STARD7, SUCLG1, TANC2, TBC1D20, TBC1D2B, TELO2, TET3, TFE3, TIMM50, TMEM106B, TMEM222, TMEM70, TMX2, TNK2, TRAF7, TRAPPC12, TRAPPC4, TRAPPC6B, TRIP13, TRPM3, TRRAP, TSEN15, TSEN2, TSEN34, TSFM, TUBA8, TUBB, TUBGCP2, TXNRD1, UBR7, UFC1, UFSP2, UGDH, UGP2, USP18, USP7, VLDLR, WDR45B, WDR62, YIF1B, YIPF5, ZMIZ1, ZNF335
